# Automated Virtual Reality Cognitive Therapy for People With Psychosis: Protocol for a Qualitative Investigation Using Peer Research Methods

**DOI:** 10.2196/31742

**Published:** 2021-10-25

**Authors:** Jessica Bond, Dan Robotham, Alexandra Kenny, Vanessa Pinfold, Thomas Kabir, Humma Andleeb, Michael Larkin, Jennifer L Martin, Susan Brown, Aislinn D Bergin, Ariane Petit, Laina Rosebrock, Sinéad Lambe, Daniel Freeman, Felicity Waite

**Affiliations:** 1 McPin Foundation London United Kingdom; 2 Research Department of Clinical, Educational and Health Psychology, Psychology and Language Sciences Faculty of Brain Sciences UCL London United Kingdom; 3 School of Psychology Aston University Birmingham United Kingdom; 4 School of Medicine University of Nottingham Nottingham United Kingdom; 5 National Institute for Health Research Nottingham Biomedical Research Centre Nottingham United Kingdom; 6 National Institute for Health Research Mindtech Co-operative Nottingham United Kingdom; 7 Department of Psychiatry University of Oxford Oxford United Kingdom; 8 Oxford Health National Health Service Foundation Trust Oxford United Kingdom; 9 National Institute for Health Research Oxford Health Biomedical Research Centre Oxford United Kingdom

**Keywords:** virtual reality, therapy, schizophrenia, agoraphobia, peer research, qualitative methods, implementation, mental health, psychosis, cognitive therapy

## Abstract

**Background:**

Many people with psychosis experience difficulties in everyday social situations. Anxiety can make life challenging, leading to withdrawal. Cognitive therapy, using active in vivo learning, enables people to overcome fears. These treatments are not readily available to people with psychosis. Automated virtual reality (VR) therapy is a potential route to increase accessibility. The gameChange automated VR cognitive therapy is designed to help people overcome anxious avoidance and build confidence in everyday social situations. A virtual coach guides the person through the treatment. Understanding user experience is key to facilitating future implementation. Peer research methods, in which people with lived experience of the issues being studied are involved in collecting and analyzing data, may be useful in developing this understanding. This encourages researchers to draw on their lived experience to explore participant perspectives and co-create knowledge.

**Objective:**

The primary objective is to use a peer research approach to explore the participant experience of a novel automated VR therapy for anxious social avoidance. This includes understanding (1) the experience of anxious social avoidance in people with psychosis, (2) the experience of the gameChange automated VR cognitive therapy, and (3) any potential impact of the therapy in people’s lives. This will inform future implementation strategies. The secondary objective is to explore how peer research can be used to co-create knowledge.

**Methods:**

Semistructured interviews will be conducted with approximately 25 people with psychosis participating in the gameChange trial (ISRCTN17308399). Participants will be recruited from the five trial centers based in National Health Service mental health trusts across England. Interviews will be conducted by two researchers. One is a peer researcher with similar lived experience to the trial participants. The other has lived experiences of mental health issues that do not directly overlap with those of the trial participants. Interview questions will focus on an individual’s experience of anxious social avoidance, experiences of participating in the gameChange VR therapy, and any changes or impact following therapy. The interview schedule was developed in collaboration with the gameChange Lived Experience Advisory Panel (LEAP), comprising 10 project advisors with lived experience of psychosis. Interpretative phenomenological analysis and template analysis will be used to explore individual accounts. The LEAP will contribute to the analysis.

**Results:**

Data collection will be conducted from April to September 2021, and analysis will be conducted from June to October 2021. As of September 28, 2021, 20 participants had been interviewed, and coding is underway.

**Conclusions:**

The study, employing a peer research approach, may provide a unique insight into the experiences of anxious social avoidance in people with psychosis and its treatment using automated VR therapy. This will inform potential future implementation of VR automated therapies in mental health services.

**International Registered Report Identifier (IRRID):**

DERR1-10.2196/31742

## Introduction

### Background

Many people with psychosis withdraw from everyday social situations due to anxiety, leaving them isolated and inactive. In a survey of 1800 people with nonaffective psychosis attending National Health Service (NHS) mental health services, two-thirds had levels of anxious avoidance comparable to agoraphobia [1]. Social avoidance has significant consequences for both mental and physical health. Although anxious avoidance is a common clinical problem, with significant negative consequences, there are no in-depth explorations of first-person accounts of anxious avoidance in people with psychosis.

The anxiety is likely to arise from several sources, such as hearing threatening or critical voices, social anxiety, paranoia, or low self-esteem. These fearful cognitions lead to avoidance or, when this is not possible, the use of in situ defense (or safety-seeking) behaviors, which prevent the receipt and processing of disconfirmatory evidence and thus maintain the anxious thought. Cognitive treatments for anxiety target the fearful cognitions and defense (or safety-seeking) behaviors [2]. Behavioral experiments provide an opportunity to test an individual’s anxious cognitions while their defenses are dropped. Overcoming anxious cognitions requires an active approach to re-evaluate fearful cognitions and generate alternative cognitions and responses in the moment. However, fearful beliefs can make this work challenging.

Virtual reality (VR; interactive computer-generated environments) provides a route to test anxious cognitions by entering simulations of feared situations. VR elicits similar cognitive and emotional responses to real-world situations (eg, [3]). However, people are aware that the computer-generated simulations are not real. This means they are more willing to enter challenging situations and experiment with alternative responses. Importantly, the learning achieved in VR then translates to the real world. VR has been used as an effective treatment for anxiety [4,5] and, more recently, as a treatment for patients with psychosis [6-8]. Automation of VR treatment [5], coupled with the use of consumer hardware, provides an opportunity to increase provision of effective psychological treatments, which are often difficult to access for people with psychosis [9].

Using a socially inclusive design process, the trial team has developed a novel automated VR cognitive treatment targeting anxious avoidance of social situations by people with psychosis: the gameChange therapy (see [10]). The 6-session VR therapy consists of computer simulations of everyday scenarios: a street, a bus, a café, a pub, a doctor’s waiting room, and a shop. Each scenario provides an opportunity to test anxious cognitions while limiting the use of defense behaviors, allowing users to gain confidence in their ability to cope. A virtual coach guides the user through the treatment. A member of staff is present to assist the user with the VR equipment and provide encouragement. As the therapy is automated, no formal training in psychological therapy is required to deliver the treatment. A multisite randomized controlled trial testing the gameChange VR therapy is currently underway in five centers in the United Kingdom (Bristol, Manchester, Newcastle, Nottingham, and Oxford) [11]. Critical to evaluating this novel treatment is understanding the participants’ experience of engaging in the therapy.

Interpretative phenomenological analysis (IPA) is a qualitative approach that focuses on understanding an individual’s lived experience and how they make sense of it [12]. IPA aims to enable the researcher to step into the participant’s world and to understand, as far as is possible, how they make sense of an experience [12]. This approach can be extended using peer-research principles, in which the research is steered and conducted by people with relevant lived experiences [13]. A peer research approach can potentially facilitate greater depth and more nuance in data collection and analysis. For example, during the interview, a shared understanding of language and empathy based on similar experiences can enhance rapport, reducing the boundaries between the researcher and the participant [14] and lessening the power balances between them [15]. This can result in the collection of more open, honest, and detailed data [15,16]. During analysis, peers bring different perspectives and insights compared to nonpeer researchers [17-19]. Working collaboratively from multiple perspectives, including a peer perspective, can enhance both the trustworthiness and the ecological validity of the analysis [17,18].

A multiple perspective design provides a structure to explore different perspectives on an experience while allowing for the homogeneity of the sample, as required for IPA [12,20]. Individual cases will be examined first. Cases of directly related groups will then be examined, such as those who have experienced the same phenomenon (in this case, anxious avoidance and the gameChange intervention). Individuals may have different perspectives on it (eg, those who had a positive experience and those who did not). This design is particularly appropriate when trying to understand “problems in the implementation and translation of effective interventions in specific social or cultural contexts” [21].

We are conducting a qualitative study using a peer research approach to explore experiences of anxious social avoidance and participation in the gameChange VR therapy. A complementary implementation study is being conducted to identify and understand issues affecting how the therapy will be adopted into health care services within the context of the trial.

### Objectives

The study has two objectives. The first is to gain an in-depth, first-person perspective on the experience of taking part in the gameChange VR therapy, which is designed to help people build confidence in everyday social situations. This includes understanding (1) the experience of anxious social avoidance in people with psychosis, (2) the experience of the automated VR therapy, and (3) any potential impact of the therapy. The second objective is to explore how peer research approaches can be used to co-create knowledge.

### Ethical Review

The gameChange trial, including this qualitative study, received Health Research Authority approval, Health and Care Research Wales approval (IRAS 256895, The gameChange Trial), and ethical approval from the NHS South Central – Oxford B Research Ethics Committee (19/SC/0075). The trial has been registered (ISRCTN17308399) and the protocol published [11].

## Methods

To increase the methodological quality and reporting, the presentation of the study will follow the guidance from the 32-item Consolidated Criteria for Reporting Qualitative Research (COREQ) [22].

### Patient and Public Involvement

There has been extensive patient and public involvement throughout the gameChange project. This includes the design and conduct of the trial as well as the development of the treatment. For example, more than 50 people with lived experience of psychosis contributed to the design of the gameChange therapy [10]. The Lived Experience Advisory Panel (LEAP), facilitated by the McPin Foundation, advises on the conduct of the project. The LEAP comprises 10 individuals with lived experience of psychosis from across the five study centers.

The LEAP has been involved in the design of this study. To date, the LEAP has contributed to developing the interview schedule, completing pilot interviews, refining the recruitment procedure, and conducting a review of all study documentation (including the information sheet and consent form) for accessibility and clarity. In response to the pilot interviews, the order of the topics was revised. The LEAP will form part of the analysis team and complete sample validation of the data.

### Peer Research Methods

This study takes a peer research approach, including employing a researcher with relevant lived experience of psychosis and social anxiety as part of the study team. This individual has not received the gameChange VR therapy but has explored the intervention to become familiar with the participants’ experience. They have been involved in developing the research proposal and designing data collection methods, they are interviewing participants, and they will be analyzing data and disseminating findings. Another researcher who has experience of poor mental health but is not a peer in this context will bring their experience of using IPA research methods and a connection to relevant experiences of mental health issues that are generic rather than specific to people with psychosis and social avoidance.

### Participants

The participants will be a subsample of approximately 25 people who took part in the gameChange trial as participants. The trial participants are people with psychosis and self-reported difficulties going outside among other people due to anxiety. There are five gameChange trial centers based in NHS mental health trusts across the United Kingdom: Avon and Wiltshire Mental Health Partnership NHS Trust, Greater Manchester Mental Health NHS Foundation Trust, Cumbria, Northumberland, Tyne and Wear NHS Foundation Trust, Nottinghamshire Healthcare NHS Foundation Trust, and Oxford Health NHS Foundation Trust. Participants for this study will be recruited from each of the trial centers. The inclusion criteria are:

- Participating in the gameChange trial and randomized to receive the gameChange VR therapy in addition to usual care.
- Willing to have the interview audio recorded.
- Willing and able to give informed consent to participate in the interview.

The project included people with a psychosis diagnosis attending NHS mental health services who experience anxious avoidance in everyday social situations. The full inclusion and exclusion criteria for participating in the gameChange trial are provided in the published trial protocol [11]. A participant may also not enter the trial if there is another factor that, in the judgement of the investigator, would preclude the participant from providing informed consent or from safely engaging with the trial procedures. Therefore, due to the COVID-19 pandemic, recruitment for people who have any of the conditions that would place them at high or moderate risk (clinically vulnerable) for a severe course of COVID-19 was suspended.

### Sampling and Recruitment

Participants will be recruited through the gameChange trial. IPA requires samples to be constructed around a conceptualized shared perspective: in this case, as outlined above, this is operationalized in terms of adults experiencing psychosis and social anxiety, in receipt of NHS mental health care, who have received the gameChange VR therapy.

Multiple perspective designs in IPA allow this homogeneity to be supplemented by some dimensions of further variability, with each conceptualized additional perspective constituting its own subsample. We are recruiting approximately 25 participants, with 3-8 participants from each of the trial centers via liaison with local trial coordinators. This is a relatively large total sample for IPA, but in this study, IPA’s commitments to depth of analysis and idiographic detail will be met via the multiple perspective design (allowing the analysis to be developed via 2-4 subsamples) and with the additional support of a template analysis component.

The primary sampling method is to invite consecutive participants as they reach the final phase of the trial. Whenever possible, this will be supplemented by purposive sampling to recruit people with a range of views and experiences of the VR intervention. This will be ascertained through therapy completion rates (low: 0-2 sessions; medium: 3-5 sessions; high: 6-8 sessions).

Sampling will also be sensitive to the demographic characteristics of the participants, aiming for a balance of gender and a range of age and ethnicity. Other dimensions of interest include the profession of the staff member facilitating VR therapy sessions (peer support worker, clinical psychologist, assistant psychologist); location of the treatment delivery (patient home, NHS clinic); and referring clinical service (early intervention; adult mental health; inpatient).

In this study, decisions about the main subsamples for the multiple perspectives design will be made at the midpoint of recruitment. This enables the design to be responsive to the key dimensions of interest emerging from the accounts. These may relate to therapy completion rate or the participants’ views of therapy. However, there may be indications that the other contextual/identity features described above are sharpening participants’ experiences of the intervention. If these are deemed more important to contrast systematically, the subsamples will be stratified accordingly.

### Recruitment Procedure

The trial coordinator at each center will facilitate recruitment. Participants will be invited to participate in the qualitative interview after the first follow-up assessment in the trial (immediately after the therapy window ends). The trial coordinator will provide potential participants with the information sheet in advance. The information sheet states that one of the researchers is a peer. The trial coordinator will introduce the participant to the researchers. The peer researcher will speak to the participant in advance of the interview. This discussion may include a further elaboration on their “peerness,” disclosing that they have some experiences in common in relation to the topic being studied, which will be documented in the research log. Written or oral informed consent will be taken at the time of the interview.

### Data Collection

Data will be gathered via semistructured interviews. Participants will be given as much choice as possible in how the interview is conducted. The primary method will be to complete the interview remotely, via video or telephone call.

There will be a primary and secondary interviewer. Participants will be told that one of the interviewers has had experiences like those being explored in the gameChange study. The other researcher, who has experience of conducting research interviews for an IPA study, will lead the first interviews. The interviews will be audio recorded. The peer researcher will lead later interviews so that the interviewee will be able to respond directly to the researcher with shared experiences, similar to the approach outlined by Harding et al (2010) [15]. The interviewer with relevant lived experience will decide when, how, and what to disclose about their peerness; this may vary according to how appropriate and comfortable this disclosure feels. Both researchers will document any disclosure of peerness in their field notes and reflect on any changes it made to the interview. These notes will be used in the analysis. At the end of the interview, the participant will be asked whether the presence of a peer researcher made any difference to their decision to participate and whether they thought it had an impact on their experience of the interview.

Peer researchers, qualitative researchers, implementation researchers, clinical psychologists, and members of the gameChange LEAP contributed to the development of the interview schedule. The development process included a review of the literature on anxious avoidance in people with psychosis, consideration of the key principles and novel features of the gameChange treatment, the complementary implementation study, and routes to include peer research methods within the interview. For example, in preparation for the interviews, the two researchers who will be collecting the data interviewed each other about their relevant lived experience. This provided potentially valuable insights into how to approach topics and ensure the phrasing was understandable and engaging. The interview schedule was further refined following pilot interviews with members of the LEAP.

The interview schedule was designed in line with IPA and phenomenological approaches [23,24], employing both open-ended descriptive and narrative questions to provide context (eg, “Tell us about what was going on in your life before you started the gameChange VR therapy?”) and descriptive/structural questions (eg, “Can you walk us through a typical gameChange VR therapy session?”). Open-ended questions are followed by individually tailored prompts to elicit further information and clarification.

### Analysis

#### IPA and Template Analysis

A total of 6 transcripts will be coded using interpretative phenomenological analysis (IPA) [12,25], and the remaining transcripts will be analyzed using template analysis [20]. A multiple perspectives design will be employed and a peer research approach will be taken, which includes collaborating with the LEAP throughout the analysis.

IPA aims to enable the researcher to step into the participant’s “lifeworld” [12] to understand, as far as is possible, how they make sense of an experience and contextualize it against the background of their lives. IPA is idiographic in that it focuses in-depth on the individual account before looking for convergence and divergence across the sample. Template analysis is a flexible approach that can be used in concert with IPA to enable larger samples to be analyzed [26]. The multiple perspectives design provides a structure for exploring both the individual case and considering cases of directly related groups [21].

IPA complements a peer research approach because it acknowledges the role of the researcher in the interpretation of the data and resulting themes. The researcher will try to make sense of the participant trying to make sense of what has happened to them. Reflexivity is welcomed. Having multiple members of the research team conduct the analysis, including those with aspects in common with the participants, will increase the depth and sensitivity of the analysis [17].

#### Analytic Method

A total of 6 transcripts will be chosen for IPA by the interviewers and the wider analysis team, based on the richness of the data and the chosen perspective (eg, participants with a positive view of the experience). The IPA will adopt analytic strategies described by Larkin and Smith (2011) [25] and Smith, Flowers, and Larkin (2021) [12]. The interviews will be transcribed verbatim. Analysis will be primarily conducted by the two interviewers, with additional input from DR, ML, SL, and FW as well as from the wider research group and the LEAP. Taking a case-by-case approach, transcripts and reflective interview notes will be read and reread. Line-by-line annotation on the participant’s claims, concerns, and understandings will be made by the two researchers. These notes require close reading and interpretation on the part of the researcher, dovetailing with the peer research approach. Annotation will identify the things that matter to each participant and the meanings associated with those things. These meanings will then be clustered and interpreted for each case. Once this process has been completed for the first interview, it will be repeated for each subsequent participant (6 cases overall). This will be used to develop themes that encompass the phenomenological experiences and understandings across the accounts of the first 6 participants. The two researchers performing the analysis will be in contact but will conduct independent analyses of the 6 transcripts. This will result in two sets of themes, one from a peer perspective.

Multiple coding can reveal new insights and explanations, especially if the researchers have different backgrounds and, therefore, different standpoints, resulting in a richer interpretation of the data [18]. The two sets of themes will be discussed and revised accordingly following review by the wider research team, including the LEAP, implementation researchers, clinical psychologists, and a qualitative research expert. The LEAP will contribute to the analysis by reviewing the preliminary themes and supporting quotes, commenting on what strikes them as interesting and the extent to which the researchers’ interpretations of the data resonate with their own [27]. Notes will be kept about any decisions made due to these meetings.

After these discussions, a provisional “template” will be agreed upon. This will be used to initiate the template analysis on the remaining data. The template evolves iteratively as more of the data are coded. Honoring the multiple perspective design, we will introduce each of the remaining groups of transcripts (based on treatment completion/uptake) one at a time, focusing on each in turn. After each group has been analyzed, we will review the template to see whether it needs to be updated as a result. We will use the steps outlined by King et al (2004) [28], namely moving systematically through the transcripts to identify data that are relevant to the research aims, assigning one or more codes from the template, and reviewing and updating the template at appropriate intervals. Once all the data have been coded and the template finalized, all transcripts will be reviewed and recoded as needed. The LEAP will again be consulted. NVivo software (QSR International) will be used to organize the data.

The result will be a set of themes (the finalized template) that represents all interviews, 6 of which will also be in case study format from the IPA. The “lamination,” or layering, made feasible by this dual approach of IPA and template analysis within a multiple perspectives design will give the study both depth and breadth. All transcripts will be deleted after the analysis is complete.

### Credibility

The four criteria for “trustworthiness” in qualitative research (credibility, transferability, dependability, and confirmability) will be monitored and addressed [29]. To increase credibility, sample validation will be conducted with members of the gameChange LEAP. The contextual constructivist position that we adopt, and the IPA methodology, assume that the data are specific to the context and time that they were collected [12,30]. However, by providing a description of this context—including information about the location (the five trial centers), participant demographic information, illustrative extracts, and situation of the findings in relation to existing literature—the reader should be equipped to evaluate how transferable the findings are to other groups and locations. Reflexive logs will be maintained throughout the research process to explore how each researcher’s background, presentation, interests, existing assumptions, and peerness have impacted the study. The logs will be used to capture initial thoughts after each interview and during the analysis process, as well as to document and justify methodological decisions. The reflexive logs, transcripts, interview schedule development, and minutes of the supervisory research team meetings will provide an audit trail of the analysis.

### Reflexivity

All researchers conducting the interviews or analysis will consider how their own backgrounds may impact data collection and analysis. This will require the researchers to reflect on the different perspectives that they are bringing to the study design, setup, data collection, and analysis. Details of the research team and reflexivity will be reported in the full manuscript in line with COREQ guidelines [22].

The research team includes a peer researcher, a nonpeer researcher, clinical psychologists who have been involved in the design and use of VR therapy, implementation researchers who are conducting a complementary study on the use of the gameChange VR therapy in mental health services, and a qualitative researcher who has pioneered the IPA approach. Therefore, existing knowledge, experiences, and hopes regarding VR therapy may impact the conduct of the interviews and analysis. We have explicitly set out to use the peer knowledge and examine peer research methods in this study. Therefore, the two interviewers will each keep a reflexive log to document the use of peerness during data collection and analysis.

### Assessing Peer Research Methods

The secondary methodological objective of this study is to learn about peer research methods and co-creation of knowledge. We will investigate the impact of peerness from overlapping, related, and nonpeer perspectives. This includes (1) how peerness is negotiated and disclosed throughout interactions with participants, (2) the effect of peerness on the interview and data collection process, and (3) the impact on analysis. This will be achieved by drawing on the researcher logs and interview field notes, evaluating participant feedback at the end of the interview (when asked about the experience of being interviewed by a peer), and discussing sections of the transcripts that document disclosure of peerness with the LEAP. To assess the impact of peer methods on the analysis, we will compare the two sets of themes resulting from the independent analysis by the two interviewers to explore key differences and similarities in the insights and interpretations. However, to identify differences based on research expertise or identity, a much larger group of peer and nonpeer coders would be required [17], which is not feasible within this study.

## Results

Data collection will be conducted from April to September 2021, with analysis performed from June to October 2021. As of September 28, 2021, data collection with 20 participants had been conducted, and coding is underway. This study is expected to conclude in 2022.

## Discussion

This protocol describes the plan for a multisite qualitative study using peer methods to explore experiences of anxious social avoidance in the context of psychosis and its treatment using an automated VR cognitive therapy program: the gameChange treatment. The gameChange VR cognitive therapy is designed to help people build confidence in everyday social situations. The qualitative study is being conducted in the context of VR therapy being tested in a multisite randomized controlled trial [11]. This study will provide insight into the individual experience of receiving novel automated VR therapy. Understanding the participant experience is likely to prove to be key to facilitating uptake in future implementation in mental health services. This understanding will be achieved using peer research methods, ensuring people with lived experience of psychosis are at the center of the research.

## Abbreviations

COREQ: Consolidated Criteria for Reporting Qualitative Research
IPA: interpretative phenomenological analysis
LEAP: Lived Experience Advisory Panel
NHS: National Health Service
NIHR: National Institute for Health Research
PPI: patient and public involvement
VR: virtual reality
